# Performance of CRASH and IMPACT Prognostic Models for Traumatic Brain Injury at 12 and 24 Months Post-Injury

**DOI:** 10.1089/neur.2022.0082

**Published:** 2023-03-01

**Authors:** Shawn R. Eagle, Enyinna Nwachuku, Jonathan Elmer, Hansen Deng, David O. Okonkwo, Matthew Pease

**Affiliations:** ^1^ Department of Neurological Surgery, University of Pittsburgh Medical Center, University of Pittsburgh, Pittsburgh, Pennsylvania, USA.; ^2^ Department of Neurological Surgery, Cleveland Clinic, Akron, Ohio, USA.; ^3^ Department of Clinical Care Medicine, University of Pittsburgh Medical Center, University of Pittsburgh, Pittsburgh, Pennsylvania, USA.; ^4^ Department of Neurological Surgery, Memorial Sloan Kettering, New York, New York, USA.

**Keywords:** adult brain injury, models of injury, traumatic brain injury

## Abstract

The Corticoid Randomization after Significant Head Injury (CRASH) and International Mission for Prognosis and Analysis of Clinical Trials (IMPACT) prognostic models are the most reported prognostic models for traumatic brain injury (TBI) in the scientific literature. However, these models were developed and validated to predict 6-month unfavorable outcome and mortality, and growing evidence supports continuous improvements in functional outcome after severe TBI up to 2 years post-injury. The purpose of this study was to evaluate CRASH and IMPACT model performance beyond 6 months post-injury to include 12 and 24 months post-injury. Discriminative validity remained consistent over time and comparable to earlier recovery time points (area under the curve = 0.77–0.83). Both models had poor fit for unfavorable outcomes, explaining less than one quarter of the variation in outcomes for severe TBI patients. The CRASH model had significant values for the Hosmer-Lemeshow test at 12 and 24 months, indicating poor model fit past the previous validation point. There is concern in the scientific literature that TBI prognostic models are being used by neurotrauma clinicians to support clinical decision making despite the goal of the models' development being to support research study design. The results of this study indicate that the CRASH and IMPACT models should not be used in routine clinical practice because of poor model fit that worsens over time and the large, unexplained variance in outcomes.

## Introduction

Traumatic brain injury (TBI) is the leading cause of death and disability for persons <40 years of age worldwide.^1^ Early identification of patients with favorable recovery trajectories is a central focus of clinical care as well as research clinical trial design. The Corticoid Randomization after Significant Head Injury (CRASH) and International Mission for Prognosis and Analysis of Clinical Trials (IMPACT) prognostic models are the most reported and validated models in the scientific literature.^2–5^ These models report a favorable performance when measured with receiver operating characteristic (ROC) area under the curve (AUC), ranging from 76% to 95%, but rarely report accuracy (4–8% of articles) or sensitivity/specificity (3–5% of articles), limiting model interpretability.^4^ More important, both CRASH and IMPACT models were developed and validated to predict outcomes at 6 months post-TBI. Since the release of these models, a growing body of research suggests that 6 months is a less relevant time point for assessing long-term TBI recovery.^2,3^

Recovery from severe TBI is a continuum of improvement.^6^ Among patients discharged from the hospital comatose, 80% regain consciousness during inpatient rehabilitation and 40% regain semi- of full-functional independence.^7–10^ The multi-center TRACK-TBI (Transforming Research and Clinical Knowledge in TBI) consortium found that outcomes for patients with moderate-to-severe TBI continue to improve from 2 weeks to 1 year post-injury.^11^ Nearly one half of patients with severe TBI recovered the ability to function independently by 12 months post-injury.^11^ We previously demonstrated that nearly 40% of patients with unfavorable outcomes at 1 year progressed to favorable outcomes by 2 years.^9^ Recognizing the vast recovery potential of severe TBI patients, we sought to extend our understanding of CRASH and IMPACT model performance beyond 6 months post-injury to include 12 and 24 months post-injury.

## Methods

### Design and participants

The current study is a secondary analysis of a prospectively collected database of severe TBI patients treated at a level 1 trauma center in [blinded for review] from November 2002 to December 2018. The database utilized includes consecutive severe TBI patients (Glasgow Coma Scale [GCS] < = 8), excluding those with age <16, pregnancy, penetrating trauma, or impending death (GCS = 3 with non-reactive enlarged pupils bilaterally).^12^ The Glasgow Outcomes Scale (GOS) was assessed at 12 and 24 months by a trained researcher. This study was approved by the University of [blinded for review] Institutional Review Board for human subjects research. Patients were fully informed before providing written informed consent to obtain access to their electronic medical health record. All data are available to qualified researchers upon request. We followed the Strengthening the Reporting of Observational Studies in Epidemiology (STROBE) checklist.^13^

### Prognostic models

#### Corticoid Randomization after Significant Head Injury (CRASH)

We utilized the computed tomography (CT) model for unfavorable outcome at 6 months and CT model for mortality at 14 days. The coefficients utilized were those validated for high-income countries.^2^ The CRASH score for unfavorable outcome and mortality was converted into percent risk for analysis to ease interpretation as described by the previously published CRASH protocol.^2,14^

#### International Mission on Prognosis and Analysis of Clinical Trials (IMPACT)

We utilized the full prediction model for unfavorable outcome and mortality at 6 months using coefficients provided by the original IMPACT publication.^14^ The IMPACT score for both mortality at 6 months and unfavorable outcome was converted into percent risk for analysis to ease interpretation as described by the previously published IMPACT protocol.

### Outcomes

Mortality was determined in two ways: 1) the patient's electronic health record or 2) a search of the Social Security Death Index. Days until mortality was operationally defined as the difference in days between date of TBI and date of death. GOS score was used to identify 12- and 24-month outcome post-TBI. Outcomes are classified from 1 to 5, where 1 corresponds to death and 5 corresponds to good recovery. Consistent with the original CRASH and IMPACT publications, we defined unfavorable outcome as a GOS score of 1–3 each time point.^2,14^

### Statistical analysis

Sample descriptive statistics were calculated and provided. We reported the AUC to identify the discriminative validity of the percent-risk models. Binary logistic regression models were built using the percent-risk models as predictors of their corresponding outcome. To assess model fit, we performed a *post hoc* analyses using the Hosmer-Lemeshow chi-squared statistic with a *p* value ≤0.05 indicating poor model calibration and *R*^2^. This statistic is important because it provides a measure of the overall precision of the model and is not often reported in studies of prognostic models for TBI.^4^ We reported overall sensitivity, specificity, positive predictive value, negative predictive value, accuracy, and false-positive rates at each time point. Statistical significance was *p* < 0.05. For all analyses, we used SPSS (v28.0.1; SPSS, Inc., Chicago, IL) and Stata software (SEv17; StataCorp LLC, College Station, TX).

## Results

We identified 598 patients who were included in the study. Our follow-up rates with complete data for GOS at 12 (84.3%; *n* = 504) and 24 months (71.4%; *n* = 427) post-trauma are consistent or better than historical norms for clinical trials.^15^ Descriptive statistics for our patient cohort can be viewed in Table 1. Overall, 153 patients in the total sample died by 14 days post-injury (25.6%), 199 died by 6 months (33.3%), 234 died by 12 months (46.4%), and 238 died by 24 months (55.7%).

**Table 1.** Descriptive Statistics for the Overall Sample Presented as Median and Interquartile Range or Count and Percentage of Sample

	Median (no.)	Interquartile range (%)
Age, years	39	25, 53.3
Days until death (*n* = 240)	8	2, 28.75
Glasgow Coma Scale	6	4, 7
Glasgow Outcome Scale at 12 months 1 2 3 4 5	234 8 104 82 76	46.4 1.6 20.6 16.3 15.1
Glasgow Outcome Scale at 24 months 1 2 3 4 5	238 2 65 63 59	55.7 0.5 15.2 14.8 13.8

### Discriminative validity and model performance of CRASH and IMPACT models for unfavorable outcomes over time

**FIG. 1.**
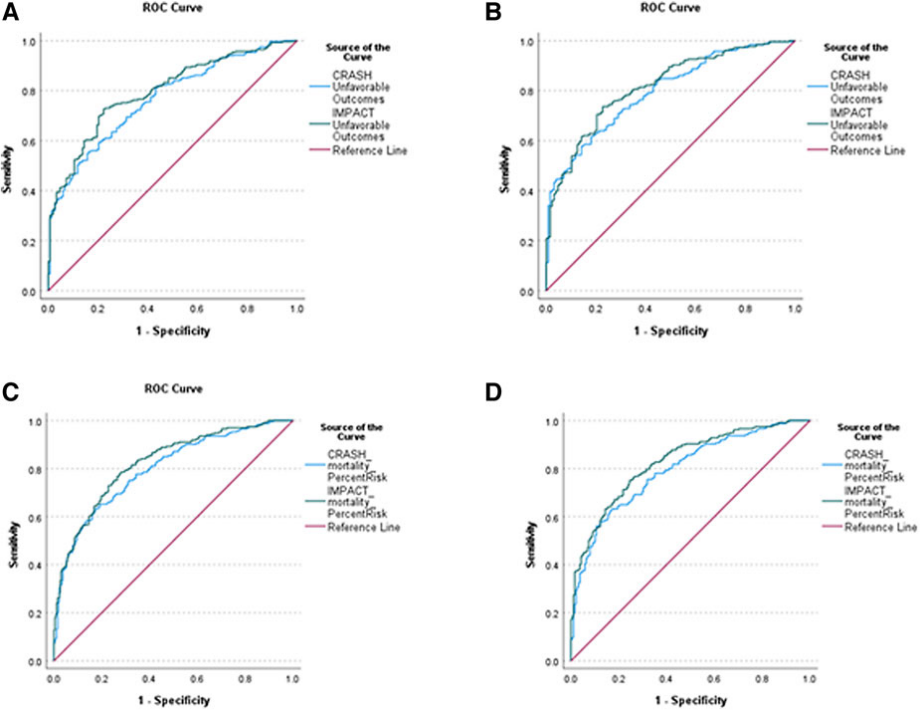
Receiver operating characteristic area under the curve outputs for CRASH and IMPACT models to predict unfavorable outcomes (GOSE = 1–4) at 12 months (**A**) and 24 months (**B**). Receiver operating characteristic area under the curve outputs for CRASH and IMPACT models to predict mortality in severe TBI patients at 12 months (**C**) and 24 months (**D**). GOSE, Glasgow Outcome Score-Extended; TBI, traumatic brain injury.

AUC and clinical characteristics can be viewed in Table 2 and Figure 1. The CRASH model had *R*^2^ values of 0.15–0.18 over time, with AUCs ranging from 0.77 to 0.80. Hosmer-Lemeshow chi-square values were statistically significant for the 12- and 24-month time points, indicating poor model calibration. Specificity for the CRASH models was poor across all time points (51.9–53.0%). Accuracy ranged from 71.5% to 74.0% with a high rate of false positives (21.4–23.9%).

**Table 2.** Model Performance Characteristics for CRASH and IMPACT Prognostic Models for Unfavorable Outcomes Over Time

	Time	R^2^	Hosmer-Lemeshow *p*	AUC	Sensitivity	Specificity	PPV	NPV	Accuracy	False positives
CRASH unfavorable outcomes	12 months (*n* = 435)	0.15	0.02^*^	0.77	82.0	51.9	76.1	60.7	71.5	23.9
	24 months (*n* = 386)	0.18	0.01^*^	0.80	84.1	53.0	78.6	62.0	74.0	21.4
IMPACT unfavorable outcomes	12 months (*n* = 435)	0.20	0.18	0.80	83.7	51.6	76.1	63.2	72.4	23.9
	24 months (*n* = 386)	0.23	0.18	0.82	85.3	53.9	78.9	64.5	74.9	21.2

^*^ Statistically significant at *p* ≤ 0.05, indicating poor model fit.

AUC, area under the curve; GOSE, Glasgow Outcome Scale-Extended; PPV, positive predictive value; NPV, negative predictive value.

The IMPACT models had *R*^2^ values of 0.20–0.23, with AUCs ranging from 0.80 to 0.82. IMPACT models also had poor specificity (51.6–53.9%), moderate accuracy (72.4–74.9%), and high rates of false positives (21.2–23.9%).

### Discriminative validity and model performance of CRASH and IMPACT models for mortality over time

AUC and clinical characteristics can be viewed in Table 3 and Figure 2. The CRASH models had *R*^2^ values of 0.20–0.21, with AUCs of 0.80. Sensitivity was moderate, with values ranging from 61.5% to 68.5%. Accuracy was moderate (70.7–73.2%), with a high rate of false positives (23.5–23.8%).

**Table 3.** Model Performance Characteristics for CRASH and IMPACT Prognostic Models for Mortality Over Time

	Time	R^2^	Hosmer-Lemeshow *p*	AUC	Sensitivity	Specificity	PPV	NPV	Accuracy	False positives
CRASH mortality	12 months (*n* = 504)	0.21	0.40	0.80	61.5	83.3	76.2	71.4	73.2	23.8
	24 months (*n* = 426)	0.20	0.74	0.80	68.5	73.4	76.5	64.8	70.7	23.5
IMPACT mortality	12 months (*n* = 501)	0.26	0.97	0.82	69.1	79.1	74.2	74.7	74.5	25.8
	24 months (*n* = 426)	0.27	0.72	0.83	76.8	73.5	78.5	71.7	75.4	21.6

AUC, area under the curve; PPV, positive predictive value; NPV, negative predictive value.

The IMPACT models had *R*^2^ values of 0.26–0.27, with AUCs ranging from 0.82 to 0.83. Sensitivity for the CRASH models was moderate across time points (69.1–76.8%). Accuracy was moderate (74.5–75.4%) with a high rate of false positives (21.6–25.8%).

## Discussion

The CRASH and IMPACT prognostic models for TBI were evaluated at 12 and 24 months post-injury in a prospective cohort of severe TBI patients. The discriminative validity for each model remained consistent over time and comparable to earlier recovery time points (AUC = 0.77–0.83).^16^ Both models had poor fit for unfavorable outcomes (*R*^2^ = 0.15–0.23), explaining less than one quarter of the variation in outcomes for severe TBI patients. The CRASH model had significant values for the Hosmer-Lemeshow test at 12 and 24 months, indicating poor model calibration past 6 months post-TBI. Despite IMPACT and CRASH maintaining AUCs through 2 years post-injury (i.e., good discriminative validity between groups), both models should not be used in routine clinical practice because of poor model fit that worsens over time and the large, unexplained variance in outcomes.

The nature of TBI is complex and heterogenous, which has hampered the ability of past work to develop accurate prognostic models. This inability is concerning, given that trauma specialists overestimate the risk for poor outcomes and underestimate the risk for favorable outcomes in patients with severe injuries, which biases decision making in early patient care.^17–19^ Previous work has demonstrated that neurosurgeons tend to be more nihilistic on long-term outcomes than general surgeons or other trauma specialists.^20^ In clinical trials in TBI, the leading cause of death is attributable to withdrawal of life-sustaining therapies, often occurring early within 72 h of injury.^21^ CRASH and IMPACT lack the discriminatory ability for individual patients to guide life-or-death decisions, given that they incorrectly predicted that nearly 1 in 5 patients would have an unfavorable outcome or die. Despite poor predictive ability, many neurotrauma clinicians do use the IMPACT or CRASH calculator to inform clinical decision making, including withdrawal-of-care decisions.^20,22^

Positive predictive value and rate of false positives may be the most important features to assess prognostic model performance given the heterogeneity in functional outcomes.^23^ Approximately 1 in 4 patients in this TBI cohort were incorrectly predicted to have unfavorable outcomes at 12 and 24 months post-injury. Lower positive predictive values are typically accompanied by higher false-positive rates, which was the case in this analysis. The high rate of misclassifying patients (∼20%) of having an unfavorable prognosis may be related to the tendency of these prognostic models to rely heavily on early post-injury clinical characteristics, which do not update based upon changes in these characteristics during recovery.^24^ Future work should investigate additional factors that may improve the positive predictive value and false-positive rate of these models.

This study has limitations. Information about withdrawal of care or cause of mortality was not available for this analysis and would have strengthened the findings. Missing data attributable to loss of follow-up could have affected the findings. We report the overall model characteristics, but certain cutoffs along the ROC curve may be clinically useful (e.g., cutoffs that maximize specificity). Though the data were prospectively collected in consecutive patients, this was a retrospective analysis, which could introduce bias. The present work was also a single-center study, and the results should be externally validated across multiple level 1 trauma centers in future research.

## Conclusion

The CRASH and IMPACT prognostic models for TBI had poor performance in key metrics for predicting unfavorable outcomes and mortality in severe TBI patients at 12 and 24 months. Positive predictive value, false positive rate, sensitivity, and specificity revealed inaccuracies associated with patient outcomes, suggesting that the current CRASH and IMPACT models are not applicable as primary predictive tools in clinical practice.

## Abbreviations Used

AUC: area under the curve
CRASH: Corticoid Randomization after Significant Head Injury
CT: computed tomography
GCS: Glasgow Coma Scale
GOS: Glasgow Outcomes Scale
IMPACT: International Mission for Prognosis and Analysis of Clinical Trials
ROC: receiver operating characteristic
TBI: traumatic brain injury
